# Caring and Working during the COVID-19 Pandemic: Perspective of Portuguese Residential Care Facility Workers

**DOI:** 10.3390/ijerph19105963

**Published:** 2022-05-13

**Authors:** Maria Miguel Barbosa, Laetitia Teixeira, Constança Paúl, Javier Yanguas, Rosa Marina Afonso

**Affiliations:** 1 Instituto de Ciências Biomédicas Abel Salazar, School of Medicine and Biomedical Sciences, University of Porto, Rua Jorge de Viterbo Ferreira, 228, 4050-313 Porto, Portugal; lcteixeira@icbas.up.pt (L.T.); constancapaul@gmail.com (C.P.); 2 Health Sciences Research Centre, Faculty of Health Sciences, University of Beira Interior (CICS-UBI), Avenida Infante D. Henrique, 6201-506 Covilhã, Portugal; 3 Center for Health Technology and Services Research (CINTESIS), Rua Dr. Plácido da Costa, s/n, 4200-450 Porto, Portugal; rmafonso@ubi.pt; 4 Aubixa Fundazioa, 20009 Donostia-San Sebastian, Spain; gerontologia@yanguas.eus; 5 Department of Psychology and Education, Faculty of Human and Social Sciences, University of Beira Interior, Estrada do Sineiro, s/n, 6200-209 Covilhã, Portugal

**Keywords:** COVID-19 pandemic, older adults, person-centered care, residential care facilities, long-term care, Portugal, healthy aging, quality of care

## Abstract

Residential care facilities (RCF) for older people are facing high demands due to the COVID-19 pandemic. The aim of this study was to explore the workers’ perspectives on the changes in work and care dynamics amidst the first wave of the pandemic at Portuguese RCF. This is a descriptive, quantitative, and cross-sectional study. An online questionnaire about pandemic-induced changes in work and care dynamics was sent to 2325 RCF. These entities were then asked to share it with their workers. The participants (*n* = 784) were mostly women (92.7%) and mostly composed of technical directors (41.6%) and direct-care workers (17.1%). The respondents reported that during the first wave of the pandemic, when compared to the pre-pandemic period, there were greater difficulties in providing care related to the basic necessities of older people (52.7%); direct-care workers were required to work more consecutive hours in each shift (69.95%); direct-care workers had to live at RCF (14.8%), and there were changes concerning the possibility of promoting person-centered care (PCC) practices. It also revealed that focusing on disease prevention and sanitary measures alone facilitates practices that reinforce the traditional model of procedure-centered care and have negative consequences on the rights and well-being of those living and working at RCF, exposing and accentuating preexisting vulnerabilities. This study considers the pandemic’s serious implications and alarming questions about basic care, dignity, living, and working conditions at Portuguese RCF. These notions reinforce the need for change through redefining care policies and practices in Portuguese RCF beyond the pandemic. The current situation provides an opportunity to adopt a formal PCC model.

## 1. Introduction

On 30 January 2020, the World Health Organization (WHO) declared an international public health emergency related to the outbreak of a new coronavirus that caused an infectious disease (coronavirus disease 2019 (COVID-19)). Given the high level of spread of this virus, in March 2020, a pandemic was declared by the WHO [1,2,3,4] COVID-19 tends to have a more severe impact, both in relation to morbidity and lethality, on people with comorbidities and preexisting health conditions—namely, cardiovascular diseases, chronic respiratory pathology, and diabetes [2,5]. Considering that institutionalized older people often have comorbidities, this group is more vulnerable to COVID-19 [6]. Additionally, long-term care facilities present an increased risk for disseminating this infection [1,7,8]. Three factors tend to contribute to this situation: residents (e.g., health vulnerabilities and dependence), professionals (e.g., shortage of workers, working in more than one location, and low literacy, regarding infection prevention and control), and institutions (e.g., shared rooms and bathrooms and agglomerations of users), creating environments with low resilience to these critical circumstances [8,9,10,11]. Considering the high risk of long-term care facilities, which makes residential care facilities (RCF) the target of extremely restrictive measures worldwide [12] robust infection prevention/control program is essential to protect residents and employees [13].

In Portugal, there are about 2500 RCF integrated in the networks of social services and facilities, with about 100,000 residents [14]. About 80% RCFs are private institutions of social solidarity (private non-profit social services, formed exclusively through the initiative of entities and supported by the social security system), and about 20% are for-profit (companies). These collective housing structures (for-profit and non-profit) for older people are required to have a license granted by the Social Security Institute, and must provide services related to food, hygiene, nursing care, recreational/occupational activities, and support for daily living activities. In Portugal, RCF are managed by technical directors who are responsible for programming institutional dynamics, as well as coordinating and supervising all employees, such as direct-care workers and nurses [15]. During the pandemic, Portuguese RCF implemented the following COVID-19 prevention and control measures: (a) distancing between people (except the necessary proximity for providing care); (b) reducing the circulation of users in the facility (e.g., keeping them in their rooms); (c) using common spaces in turns; (d) reorganizing daily activities/ceasing ludic collective activities; (e) separating care workers into teams to assist specific groups of users; (f) prohibiting the entry of companions and isolating new residents for at least 14 days in every new admission; and (g) isolating residents for no less than 14 days whenever they leave the institution (less than 24 h) [5,16]. Visits were suspended in March 2020 [5] and resumed on May 18, subject to requirements, such as scheduled and limited time slots, specific and controlled visitation areas, physical distancing, and the use of masks [17].

The pandemic context might generate changes in the quality of gerontological care and promote standardized practices that can reinforce the traditional care model [8,18,19]. This traditional care model, also known as sanitary or biomedical, tends to focus on task efficiency, standardized procedures, the deficits of the older people, and biological aspects of illnesses. Before the pandemic, the traditional care model had already shown signs of exhaustion and negative impacts on older people—namely, the violation of their rights and self-determination [20,21]. Therefore, applying this model may leave RCF vulnerable in critical contexts. Meanwhile, person-centered care (PCC) has the potential to lessen this negative impact and is currently recognized as the highest standard of quality in the care of older people (with positive repercussions on the well-being of both residents and care workers) [22,23]. PCC is an ethical paradigm of interaction that is based on respect for the rights of older people; it places the individuals at the center of the care dynamics, views them as unique beings, delivers personalized practices, enables them to play an active role as effective decision-makers, responds to their needs, and promotes their autonomy and potentialities [20,24].

Scientific studies on the formal applications of PCC in RCF are scarce in Portugal, while studies and results that are congruent with the application of the traditional care models are evident. For example, an investigation into the quality of Portuguese RCF that involved the analysis of 3685 complaints found that inadequate care was provided and determined that the systems used to evaluate the quality of care were based only on the quality standards of procedures and tasks [25]. Unfortunately, in Portugal, the violation of rights and the generalized devaluation of self-determination for older people residing in RCF continues to be identified [25,26,27], and changing the current traditional care model is greatly needed. In this sense, considering the pandemic context, it is necessary to characterize the present moment and understand how it can improve care and enhance its quality during and beyond the pandemic. Therefore, the aim of this study was to explore the perspectives of workers on pandemic-induced changes in work and care dynamics during the first wave of the COVID-19 pandemic at Portuguese RCF. Specifically, it will explore whether (a) the dynamics of work and care have changed, (b) whether there have been differences in the ability to respond to older peoples’ basic needs, (c) whether there have been changes in the opportunity to promote practices of PCC for older people, and (d) whether care has become more sanitary and less centered on older people.

## 2. Materials and Methods

### 2.1. Study Design

This exploratory and descriptive study is quantitative and cross-sectional. The online data for the study were collected between 8 July and 18 October 2020.

### 2.2. Sample

The study addressed workers employed at Portuguese RCF for older people. Participants’ inclusion criteria were: having worked at the organization during the first lockdown (March/April 2020), having worked at the organization since at least January 2020 (3 months before the pandemic), and accepting the commitment to participate in the study. Not working at the RCF at the time of data collection was an exclusion criterion.

All RCF for older people in mainland Portugal and the islands (Madeira and Azores) were contacted and invited to participate in the study. The contact database included the following: (a) for mainland Portugal, all formal email addresses (*n* = 2250) present in the Carta Social (the official information on social services institutions in operation in mainland Portugal—the Office of Strategy and Planning of the Ministry of Labor and Social Security of the Portuguese Republic); and (b) for the Azores and Madeira archipelagos; all formal email addresses (*n* = 75) were obtained through the corresponding Social Security Institutes and/or the official online pages of social facilities (present in Google Maps listings). This process comprised sending 2325 emails. In addition, the researchers used the social network pages (namely Facebook) of their universities, research units, and personal, to disseminate the study and involve more participants. The contact with RCF included a description of the project, objectives, the link to access the questionnaire in Google Forms, and a request to share the link with the institutions’ staff members (meaning that there could be several responses per institution).

### 2.3. Protocol

The global protocol had an average response time of 4 min and consisted of 37 questions organized into four parts: sociodemographic component, description of the institution (type of organization, i.e., for-profit/non-profit, number of users and generic geographical area), characterization of changes in work and care dynamics (which required workers to compare two different periods pre-pandemic and current pandemic period), and a questionnaire on the perception of workers regarding the possibility of promoting PCC for older people during the pandemic. Despite not having an incidence or prevalence objective, the study included questions related to the existence of COVID-19 cases at the participant’s RCF to better characterize their contexts.

#### Questionnaire on the Perception of Workers Regarding the Possibility for Promoting PCC Practices at RCF

Instruments related to PCC applied at RCF are not yet available in Portugal, and no measurement tool was found to assess the effect of a pandemic or to compare between before and after a critical event in these settings. Owing to the urgent need to explore the pandemic-induced changes in care dynamics in these facilities, this study required the development of a specific tool that would serve this objective and could be used to collect the perceptions of workers on the field. The methodology that best fitted the purpose was a content-related approach. Therefore, the questionnaire was built on the basis of systematizing the state of the art regarding PCC provided to older people (e.g., [21,28]). This process allowed for a conceptual definition and served as a guide for elaborating a set of items that were submitted for analysis by a committee of experts to ensure the validity of the content. Subsequently, the questionnaire was subjected to a pilot test with five workers pertaining to the target population. This procedure allowed us to analyze whether the instructions, items, and response options were clear and understandable [29]. According to the suggestions resulting from the pilot test, and with the assistance of the committee of experts, the necessary adjustments were made to optimize the questionnaire [29].

The final questionnaire consisted of 22 statements about PCC practices that referred to the general care provided by the workers’ institutions as a whole. For each of the statements, the respondents were asked to indicate whether the possibility of promoting these practices was “increased”, “maintained”, or “reduced”, when compared to before the pandemic. The analysis of this questionnaire’s internal consistency was carried out using Cronbach’s alpha, which was 903.

### 2.4. Data Analysis

A statistical analysis was performed using IBM SPSS Statistics 26 (Armonk, NY, USA). Regarding the exploratory nature of the study, a descriptive analysis of the data were performed using measures of central tendency (mean or median) and dispersion (standard deviation [sd] or interquartile range (IQR) or absolute and relative frequencies.

### 2.5. Ethical Aspects

This research is part of the project: “Atenção Centrada na pessoa na prestação de cuidados na velhice: abordagens e instrumentos de avaliação” approved by the Ethics Committee at the Universidade da Beira Interior (nº CE-UBI-Pj-2019-057-ID1555) with an addendum authorized on 21 July 2020, for the study: “Pandemia COVID-19 e cuidados em Estruturas Residenciais para Pessoas Idosas”.

The link through which participants were able to access the questionnaire contained informed consent, along with the context of the study, the objectives, voluntariness, guarantee of confidentiality, and the availability of an investigation team member (for contact and clarification).

## 3. Results

### 3.1. Characterization of the Study Participants

Table 1 presents the characteristics of the sample under study (*n* = 784). The participants were between 19 and 71 years old (mean = 38.19 years, sd = 9.11 years) and were mostly women (92.7%, *n* = 727). The median time of work in the area of providing care to older people was 108.00 months (IQR = 129.00 months). The majority of the professionals (79.5%, *n* = 623) reported working at private institutions of social solidarity (private non-profit social services); the number of users from the organizations where they were employed ranged between 4 and 154. The main questionnaire respondents were technical directors (41.6%, *n* = 326), direct-care workers (17.1%, *n* = 134), and nurses (10.5%, *n* = 82). Regarding geographical distribution, the responses were organized according to the division adopted by the Direção-Geral da Saúde.

### 3.2. Characterization of Changes in Work and Care Dynamics during the Pandemic

Comparing the number of workers providing direct care before and during the first wave of the pandemic at the institutions where the participants worked, 51.4% (*n* = 403) of the sample indicated that this number was maintained, 28.4% (*n* = 223) of the sample reported that there were fewer workers providing direct care during the pandemic, and 19.8% (*n* = 155) reported that the number of workers increased. Considering the number of hours worked in each shift by direct-care workers at their institutions during the first wave of the pandemic (compared to before the pandemic), 69.95% (*n* = 547) of participants reported that the shifts had more consecutive hours, 26.3% (*n* = 206) stated that the shifts had the same number of hours, and 3.7% (*n* = 29) indicated less hours per shift. Regarding the accommodation of direct-care workers during the first wave of the pandemic, 85.1% (*n* = 667) of the participants reported that they slept in their own homes, and 14.8% (*n* = 116) stated that they were housed at the institution. Compared to before the pandemic, responding to the basic needs of older people (e.g., hygiene and food) during the pandemic was considered more difficult by 52.7% of the participants (*n* = 413), the same by 43.2% (*n* = 339), and easier by 4% (*n* = 31). The majority of participants (81.8%, *n* = 641) revealed no COVID-19 infection cases at the organizations where they worked. Out of the 18.1% (*n* = 142) of workers who reported the existence of COVID-19 cases, the existing cases were among workers and users (9.3%, *n* = 73), only workers (6.3%, *n* = 49), and only users (2.8%, *n* = 22). In 6.4% (*n* = 50) of the sample, there were deaths from COVID-19 reported at the institutions.

### 3.3. Characterization of the Possibility of Promoting PCC during the Pandemic

Respondents classified the possibility of promoting PCC practices at their work institutions as a whole during the pandemic (compared to before it) according to their own perceptions, which is shared in Table 2. Most responses indicated that the institutions maintained the possibility of: individualizing care (i.e., adapting care practices to the characteristics of each resident) (item 1), centering attention on the user (rather than on sanitary procedures/tasks) (item 2), giving users the possibility of making decisions (item 3), maximizing the potentialities of each user (regardless of age, limitations, and pathologies) (item 5), promoting the autonomy of users (item 6), respecting users’ rights (item 7), allowing users to use their personal property (e.g., watches, jewelry, and other objects) (item 8), taking care of users’ personal appearances according to their preferences (item 9), taking into consideration the life story of each user in their care plan (item 10), keeping routines flexible according to the desires of users (e.g., hygiene schedules and feeding) (item 12), taking time to provide individualized care (item 13), ensuring users’ privacy (item 15), working among other professionals (item 21), and workers putting themselves in the place of older people (item 22).

From the perspective of the majority of participants, the pandemic situation reduced the possibility of promoting interactions between the users (item 17), involving the family and other significant people in users’ care (item 19), and promoting the interaction and bonds among users and their families and/or people close to them (item 20).

According to most responses, the chances of promoting any of the PCC practices at the institutions where the participants worked did not increase during the pandemic. Nevertheless, assigning the same workers to the same users (i.e., reducing turnover) (item 11), working among other professionals (item 21), and workers putting themselves in the place of older people (item 22) were the most frequent practices.

## 4. Discussion

This study aimed to explore the perspectives of workers concerning pandemic-induced changes in work and care dynamics during the first wave of the pandemic in Portuguese RCF. The first objective of this study was to explore whether the dynamics of work and care had changed. The results show that the number of workers during this phase tended to remain the same or decrease in a significant part of the sample. This result is worrying and contrary to the argument put forth by Pitkälä [11] and Armstrong et al. [30] that the number of professionals with experience and training should increase at RCF in times of crisis, such as the current one. The number of workers should increase because the pandemic exposes the general RCF workers to greater overload in regard to work (e.g., application of new procedures and increased surveillance), health (e.g., risk of infection), personal issues (e.g., managing family life owing to the closure of services such as schools), emotional issues (e.g., dealing with the unknown and the fear of becoming infected/infecting others), social issues (e.g., stigma), and ethical issues (e.g., managing restrictions) [11,31,32,33]. Consequently, an unchanged or decreased number of workers during the pandemic may suggest an increase in the vulnerability of systems, and that the remaining human resources have had to deal with the increased overload and negative impacts on their working conditions. Worryingly, most participants reported that direct-care workers at their institutions worked more consecutive hours for each shift, which corroborates, for example, the data presented by Ayalon et al. [31]. These results may suggest that workers were further overloaded during this pandemic phase. Additionally, more than 100 respondents in this study reported that direct-care workers were housed at their institutions. This practice may have advantages from the point of view of protecting the health of older people (as it minimizes contact with the outside world). However, according to Ayalon et al. [31] this is a violation of workers’ rights and working conditions. As working conditions are also conditions of care, these results may have serious consequences for the well-being of workers and residents at RCF [31]; therefore, more attention must be paid to working conditions at Portuguese RCF, especially during the pandemic.

The aforementioned data may be related to the results found regarding the second objective of this study—to determine whether differences exist concerning the ability to respond to basic needs during the pandemic, compared to before the pandemic. Most of the study participants indicated a greater difficulty, thereby revealing a reduced quality in basic care. This raises alarming questions about living conditions and dignity at Portuguese RCF during the pandemic. Furthermore, as argued by Ayalon et al. [31] and Gardner, States, and Bagley [34], in the absence of external visitors, which is the case for Portuguese RCF, the care and living conditions of residents are less monitored, which can expose them to the risk of negligence and ill-treatment.

Regarding the third objective—determining whether there have been changes in the opportunities to promote PCC practices compared to before the pandemic—most of the respondents indicated that the possibility of promoting about half of the PCC practices presented in this study had been maintained. Considering that traditional/sanitary care approaches prevail in Portugal, this result may suggest that the opportunity has not changed since it could not be identified even before the pandemic (which frames the workers’ mindsets and the care they provide in practices that are focused on procedures).

The results showed a clear reduction in the possibility of promoting interactions between users, involving families/other significant people in providing care to users, and promoting interactions and bonds between users and their families/people close to them. These results may indicate the consequences of the measures taken to ensure infectious control, such as those previously mentioned in this document (e.g., [5,16,17]). The restrictive measures applied triggered ethical dilemmas [12,32]. While these measures play an important role in infectious disease prevention/control, they also have worrying effects [8,19,35,36] as they promote standard practices, tend to focus on procedures, and generally constitute a threat to the practice of the PCC paradigm. Dichter et al. [8] stated that the restrictions and social isolation measures are the opposite of PCC. This leads to the exploration of the fourth objective of this research. According to the PCC perspective, the general restrictive measures are a threat to the rights (e.g., self-determination), will, and wishes of institutionalized older people [19,33], which are essential values in PCC. Moreover, (a) social distancing and restricting physical contact reduces the opportunity for warm-hearted treatment and increases loneliness (which is directly related to psychological suffering and a decline in physical and cognitive functioning) [8,12]; (b) using personal protection equipment can lead to fear and anxiety [37] which are barriers to communication and increase the difficulties related to sensory deficits, making the context less accessible to the older people; (c) restricting their mobility within the facility or their ability to exit to the outside world challenges the autonomy and functionality of older people [12] and reduces the possibility of them managing their own daily lives; (d) using common spaces in shifts and terminating group activities reduces the opportunities to establish bonds, commitments, and a sense of belonging to a group; (e) prohibiting the entry of relatives at the time of admission decreases the support and follow-up during older people’s integration (e.g., the possibility of sharing personal fears) [8] and (f) suspending/adjusting visits restricts the support of social ties and the sense of connection, significance, and belonging [38], as well as the continuity of the life story [39]. In addition, managing the end-of-life psychological, social, and spiritual needs of older people also becomes more difficult [8,32]. In general, the adopted measures relate to the worsening of loneliness in older people, which can have serious effects on their physical and mental health [8,11].

In light of the overall results, the pandemic-induced changes perceived by workers may be evidence of pre-existing vulnerabilities at RCF, such as unsustainable care practices (e.g., those who are more focused on procedures and task efficiency and less focused on older people and workers’ rights and well-being) and insufficient resources (e.g., a low ratio of workers to users and low valuation of workers). This analysis is congruent with entities and authors who report that the risks observed during the pandemic “are not new” ([19], p.16), and that this crisis has revealed and exacerbated pre-existing vulnerabilities in RCF care [11,31,33,40]. Therefore, the results are relevant to better understand the impact of the pandemic (and help gerontological services, workers, and policy makers to deal with this worldwide situation). The exploration of this critical moment can also serve to establish a starting point from which to guide the evolution of care models at Portuguese RCF beyond the pandemic.

### Limitations, Strengths, and Future Research

This study provided empirical insights based on quantitative data and featured participants from all health regions of mainland Portugal and the islands. As the absolute confidentiality of the data were guaranteed and there could be several responses per institution, it was not possible to identify the different participating institutions and determine response rates. The data were collected in a specific period (during the first wave) and must be contextualized accordingly. It should be noted that the asymmetric pandemic development in the country can cause RCF to be in different phases of pandemic management, and this may have influenced the results. Considering that 81.8% of the participants revealed no cases of COVID-19 infection in their organizations, the workers who volunteered to participate in this study were probably less burdened at the time of the inquiry and their routine activities were probably less disrupted than those in other RCF. Additionally, online data collection may have hindered the participation of more workers—namely due to low digital literacy and utilization. Taking the study’s nature into account, the results obtained should be considered prudently, as they may be influenced by the social desirability associated to the replies. It would be beneficial to complement these results with the perspective of older people, which was not contemplated in this study due to the impossibility of accessing that target population during this period. As such, the perspectives of care recipients at RCF are very important and must be considered in future studies. Considering the pandemic evolution and its enhanced effect on RCF, it seems critical to study how the organization of care, the management of work, and care models could be redesigned to make them more sustainable, safer (e.g., in terms of infectiousness), and person-centered.

Future research is necessary in the field of PCC at RCF, namely in the scope of developing and validating measurement tools that contemplate several sources (e.g., workers, older adults and their family members); creating guidelines for employing PCC; testing any changes to care practices by assessing the impact of such changes on the well-being of older adults receiving care, as well as the well-being of care workers; and the cost-effectiveness of this care paradigm for the organizations.

## 5. Conclusions

The results of this descriptive study reflect the pandemic-induced changes perceived by Portuguese RCF workers during the first wave of the pandemic. Changes in the dynamics of care and work were observed and the pandemic context has shown potentially serious implications for those who live and work in these facilities. These changes may have important consequences on the quality of basic care and exacerbate the violation of residents’ rights, which raises alarming questions about the well-being, dignity, and living conditions in this context. While the surveillance of older people at RCF is fundamental during the pandemic, focusing on disease prevention and sanitary measures (and less on older people and their psychosocial needs, choices, and potentialities) accentuates and legitimizes more standardized care practices that may unfortunately reinforce the traditional sanitary model, which was already showing signs of exhaustion and negative impacts on older people before the pandemic. Therefore, the pandemic context threatens the sustainability of systems that expose and accentuate pre-existing vulnerabilities, in terms of human, physical, and psychosocial resources.

These conclusions demonstrate why an immediate intervention is required. It is critical to analyze, minimize, and compensate for the existing consequences by preparing measures that offset the damage caused by the pandemic and ensure a transition to recovery by promoting resources and support for the mental and physical well-being of those who live and work at RCF. This study also reinforces the need for change through the redefinition of policies and practices and provides information that serves to guide the evolution of care models at Portuguese RCF beyond the pandemic. Consequently, this crisis promotes the potential for change, as it sets up an opportunity to reflect, replan, and claim a new model of residential care for older people based on the PCC paradigm. This requires research and replanning in the short, medium, and long term, so that the links between science and policy actions are strengthened.

This study aims to contribute to scientific advances in this area, placing science at the service of practice during a pandemic crisis, so that policy makers, services, professionals, older people, and other entities have more information and resources to make wise decisions; deal with new challenges; enhance working and living conditions; promote healthy aging, with more respect for human rights, dignity, and quality of life at RCF.

## Figures and Tables

**Table 1:** Sociodemographic and professional characteristics of the participants (*n* = 784).

	*n*	*n* (%)/Mean (sd)/Median (Interquartile Range, IQR)
Age (range: 19–71)	768	M = 38.19 (sd = 9.11)
Sex	780	
Female		727 (92.7%)
Male		53 (6.8%)
Schooling	780	
Between 1 and 4 years		4 (0.5)
Between 5 and 7 years		2 (0.3)
Between 8 and 9 years		31 (4.0)
Between 10 and 12 years		99 (12.6)
More than 13 years		644 (82.1)
Months of work in the area of older people care (range: 7–480)	784	Mdn = 108.00; IQR = 129.00;
Type of organization management	769	
Private institution of social solidarity (private non-profit social services)		623 (79.5)
>Private (company)		>146 (18.6)
Number of users [range: 4–154]	726	Mdn = 50; IQR = 38
Professions	783	
Technical director		326 (41.6%)
Direct-care worker		134 (17.1%)
Nurse		82 (10.5%)
Animator		68 (8.7%)
Social worker		33 (4.2%)
Psychologist		30 (3.8%)
Others (e.g., physiotherapist, speech therapist, gerontologist, occupational therapist)		110 (14%)
Geographical area	781	
North		193 (24.6%)
Center		307 (39.2%)
Lisbon and Tejo Valley		154 (19.6%)
Alentejo		49 (6.3%)
Algarve		27 (3.4%)
Azores		22 (2.8%)
Madeira		29 (3.7%)

**Table 2:** Possibility of promoting PCC practices during the first wave of the pandemic (compared to before the pandemic).

	*n* (%)		
	Increased	Maintained	Reduced
1. Individualizing care (i.e., adapting the care to the characteristics of each user) (*n* = 770)	204 (26.0%)	**456 (58.2%)**	110 (14.0%)
2. Centering attention on the user (rather than on sanitary procedures/tasks) (*n* = 768)	205 (26.1%)	**387 (49.4%)**	176 (22.4%)
3. Giving users the possibility of making decisions (*n* = 770)	44 (5.6%)	**417 (53.2%)**	309 (39.4%)
4. Meeting the wishes and desires of users (*n* = 770)	112 (14.3%)	348 (44.4%)	310 (39.5%)
5. Maximizing the potentialities of each user (regardless of age, limitations, and pathologies) (*n* = 773)	118 (15.1%)	**473 (60.3%)**	182 (23.2%)
6. Promoting the autonomy of users (*n* = 772)	89 (11.4%)	**446 (56.9%)**	237 (30.2%)
7. Respecting users’ rights (*n* = 772)	63 (8.0%)	**596 (76.0%)**	113 (14.4%)
8. Allowing users to use their personal property (e.g., watches, jewelry, and other objects) (*n* = 768)	29 (3.7%)	**596 (76.0%)**	143 (18.2%)
9. Taking care of users’ personal images and appearances, according to their preferences (*n* = 775)	64 (8.2%)	**547 (69.8%)**	164 (20.9%)
10. Taking into consideration the life story of each user in their care plan (*n* = 772)	43 (5.5%)	**642 (81.9%)**	87 (11.1%)
11. Assigning the same professionals to the same users (i.e., reducing turnover) (*n* = 771)	270 (34.4%)	375 (47.8%)	126 (16.1%)
12. Flexibility of routines according to the desires of users (e.g., hygiene schedules and feeding) (*n* = 774)	79 (10.1%)	**490 (62.5%)**	205 (26.1%)
13. Taking time to provide individualized care (*n* = 772)	94 (12.0%)	**465 (59.3%)**	213 (27.2%)
14. Taking time to talk with the older people (*n* = 773)	170 (21.7%)	361 (46.0%)	242 (30.9%)
15. Ensuring users’ privacy (*n* = 777)	68 (8.7%)	**656 (83.7%)**	53 (6.8%)
16. Promoting activities of interest to users for them to engage in throughout the day (*n* = 774)	109 (13.9%)	**376 (48.0%)**	289 (36.9%)
17. Promoting interactions between users (*n* = 774)	85 (10.8%)	311 (39.7%)	**378 (48.2%)**
18. Promoting proximity and warm treatment among care workers and users (*n* = 771)	120 (15.3%)	366 (46.7%)	285 (36.4%)
19. Involving the family and other significant people in users’ care (*n* = 768)	51 (6.5%)	191 (24.4%)	**526 (67.1%)**
20. Promoting the interaction and bonds among users and their families and/or people close to them (*n* = 772)	121 (15.4%)	246 (31.4%)	**405 (51.7%)**
21. Working among other professionals (*n* = 777)	324 (41.3%)	**376 (48.0%)**	77 (9.8%)
22. The professionals put themselves in the older people’s place (item 22) (*n* = 772)	275 (35.1%)	**454 (57.9%)**	43 (5.5%)

## Data Availability

Not applicable.
